# Web Analytics to Objectively Measure the Success of Educational Resources in Human Anatomy

**DOI:** 10.1007/s40670-025-02304-z

**Published:** 2025-02-06

**Authors:** Pilar Alberola-Zorrilla, Rosa Zaragozá-Colom, Amparo Gimeno-Monrós, Daniel Sánchez-Zuriaga

**Affiliations:** 1 Research Group on the Clinical Anatomy of the Musculoskeletal System (GIACAL), Department of Anatomy and Human Embryology, Faculty of Medicine, Universitat de València, Av. Blasco Ibáñez 15, 46010 Valencia, Spain; 2 Teaching Innovation in Anatomy (INNAt) Consolidated Teaching Innovation Group, Department of Anatomy and Human Embryology, Faculty of Medicine, Universitat de València, Av. Blasco Ibáñez 15, 46010 Valencia, Spain

**Keywords:** Engagement, E-learning, Instagram, Google Analytics, Web analytics

## Abstract

Traditional human anatomy teaching relies on face-to-face models, although new methodologies based on virtual platforms can enhance autonomous learning. However, the specific type of online tools preferred by the students remains unclear. This study compared student engagement with two teaching tools: (1) online unidirectional lessons and (2) an interactive Instagram profile, @eldeanato. Both tools incorporated new anatomical illustrations and detailed dissection photographs. The Instagram profile included questionnaires via the Instagram stories quiz sticker. Students’ satisfaction was assessed by means of online surveys. Engagement with each of the interventions was evaluated using objective measures of web analytics: percentages of interaction with the questionnaires on Instagram and adding Google Analytics tools to the online lessons’ website code. Student satisfaction with both types of material was high, but the participative material had much higher engagement scores. The Instagram profile allowed continuous participation, with increasing followers and interaction levels. Our results show the importance of objective engagement measurements to evaluate the success of innovative teaching interventions. In order to increase student adherence, teaching interventions should be participative and integrated with popular social networks, like Instagram, even though this is a field that could be seen as estranged from academic teaching.

## Introduction

The use of social media in education presents challenges for educators. The lack of control over the accuracy of the contents is a significant issue due to the presence of “fake news” or inaccurate information [1]. Moreover, the use of social media has been linked to increased anxiety, depression or other mental health problems, among students, in the context of learning [2]. Despite these challenges, the current generation of 2024 higher education students has grown up with social media [3]. Most higher education students do not only use social media for leisure purposes, but also as a tool for acquiring and sharing information, as well as communicating with their colleagues on study-related topics. This happens across the entire spectrum of health sciences careers, from prospective medical students [4] to pharmacy [5], dental education [6] or radiation therapy [7]. Consequently, university teachers have increasingly recognized the benefits of using social media as an educational tool [8, 9]. The use of social media allows university teachers a greater dissemination of information, as a single post can reach hundreds of students globally, a feat that traditional passive dissemination methods cannot achieve [10]. Thus, social media platforms such as Facebook, YouTube, and Twitter have been used by teachers as a support to their lectures [11–13].

Several studies have examined the advantages and disadvantages of the use of social media in medical higher education [12, 13]. These studies list some problems and barriers to the use of this type of tools, such as generational gaps and unfamiliarity, distraction and disruption, overreliance, the lack of quality assurance, information overload and the lack of privacy and professionalism. But in general, studies reporting evaluative outcomes show mostly positive feedback from students [14]. Beneficial effects include increased active collaborative learning through engagement with content posted on social media, enhanced student participation, communication and commenting among pairs, as well as improved access to resources regardless of students’ location. There are differences in the use of social media between medical students and teachers. Whereas teachers value traditional content such as videos, articles and explanatory comments, students are thought to place greater value on posts containing interactive material [11].

Despite doubts about the specific type of resources preferred by the students, anatomy courses have embraced the use of social media as a teaching aid [15]. Anatomy is a subject that relies heavily on the visual understanding of concepts. For that reason, many anatomists have created successful profiles on image-based platforms like Facebook, which students perceive as valuable study tools [16, 17]. Twitter profiles devoted to neuroanatomy, which is often perceived as heavy going and difficult to study [18], have also been used and highly valued by students. The use of Twitter in the context of neuroanatomical teaching appears to increase student engagement and improve their perception of the subject, leading to increased adherence to studying [19].

Generation Z students present a new challenge for the educational use of new social media platforms, as they seem to favour platforms that offer interactive engagement, rather than traditional passive content consumption [1]. Instagram has emerged as their preferred social media platform, but the use of Instagram in anatomical teaching raises some concerns. It may seem that the use of a leisure-focused social media platform like Instagram could compromise the quality of the contents, due to its a *priori* distant connection to academic teaching. In this sense, quality open-access, traditional and more passive contents, shared online and prepared under the supervision of the teaching staff, could be seen as more reliable and thus offer a greater appeal to students. Nowadays, students have access to a wide range of anatomy materials of various types, both passive and active. However, we lack objective data about differences in the level of engagement of students with both types of resources, apart from the results of the usual satisfaction surveys completed after educative innovation projects. We are unaware of students’ preferences.

The objective of this study was to compare the level of engagement of the students with two different innovative teaching tools: online lessons with open-access high-quality anatomical images covering the content of human anatomy courses (referred to as “unidirectional” or passively assimilated material) and an Instagram profile based on that same images which allowed students interaction by means of quizzes posted on the profile stories (referred to as “interactive” or actively assimilated material). In addition to evaluating the degree of student satisfaction with both interventions through satisfaction surveys, the level of engagement with these two types of resources was objectively assessed by means of web analytics tools.

## Material and Methods

### Participants

Each academic year, first-year medical students at the University of Valencia are divided into four groups. All of them must take a subject on human musculoskeletal anatomy in the first semester. The syllabus of this subject is identical for the four groups, and the distribution, calendar and evaluation of the contents are previously agreed upon by all the professors responsible for the subject. Out of those four groups, two groups of first-year medical students participated in this study during the 2022–2023 academic year, one with 76 enrolled students (the unidirectional intervention group) and another with 83 (the interactive intervention group). There were no differences between the groups in terms of gender composition, age distribution or access conditions to medical studies. Both interventions were based on the contents of upper limb anatomy in the human musculoskeletal anatomy subject.

### Ethical Approval

Approval for this study was obtained from the university Consulting Committee on Teaching Innovation Proposals, with approval number/ID UV-SFPIE_PID-2078036.

### Types of Interventions

#### Unidirectional Material

Online lessons focused on a specific aspect of the first-year anatomy course: the osteology and myology of the upper limb. The contents underwent a thorough review by different professors from the Department of Anatomy, who deemed it interesting and of high quality.

**Fig. 1:**
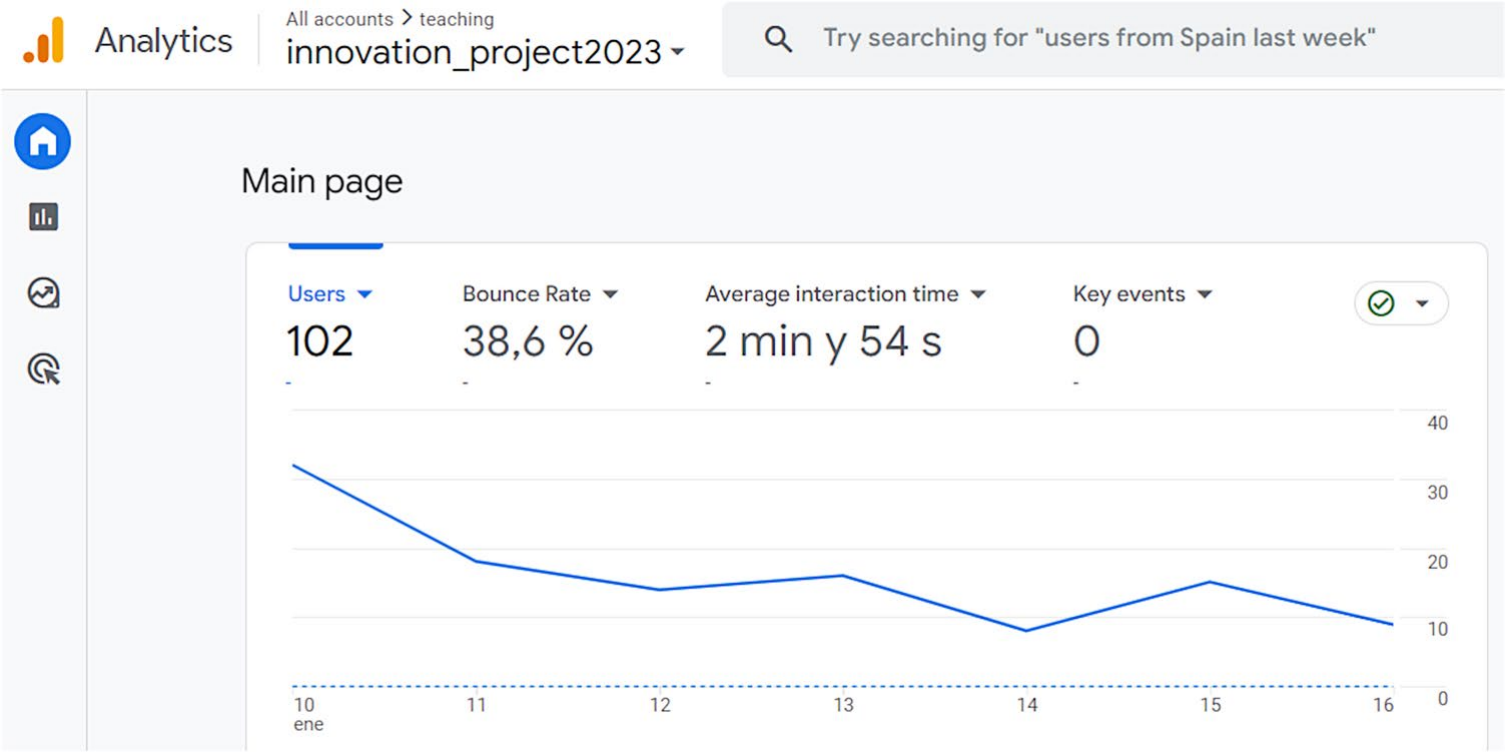
Main page of the project’s Google Analytics account, showing the web analytics variables corresponding to the unidirectional website engagement results

Lessons took the form of a website, created with eXeLearning [20], an open-source tool designed for the development of online educational content. To objectively assess the success of this intervention, the resulting HTML code was supplemented with the website traffic statistics tools of Google Analytics [21] (Fig. 1). Online lessons contained new original anatomical images, distributed under a CC BY-NC-ND license. They were generated from photographs of real structures, obtained from a careful dissection specifically conducted for this project. Additionally, new illustrations were created by an anatomical illustrator.

A link (www.uv.es/sanzuda) was provided to the 76 students enrolled in the target group via e-mail, a week before the final exam of the anatomy course, to ensure those students would be actively studying the subject. It was assumed that, anxious about the proximity of the exam, students would more likely turn to material provided by the course’s teaching team as a study aid, and if they found it useful, they would share it with their classmates. The e-mail was sent by the professor responsible for the project, who was a member of the Department of Anatomy but did not have any teaching or scoring responsibilities in the target group. This was done in order to avoid biases in the degree of use of the material, due to the students having the impression that the material provided was somehow mandatory study or that it was more likely to be asked in the exam of the subject. The e-mail explicitly informed the students that their grades would not be influenced whatsoever by their use or the lack of use of the provided material.

#### Interactive Intervention

**Fig. 2:**
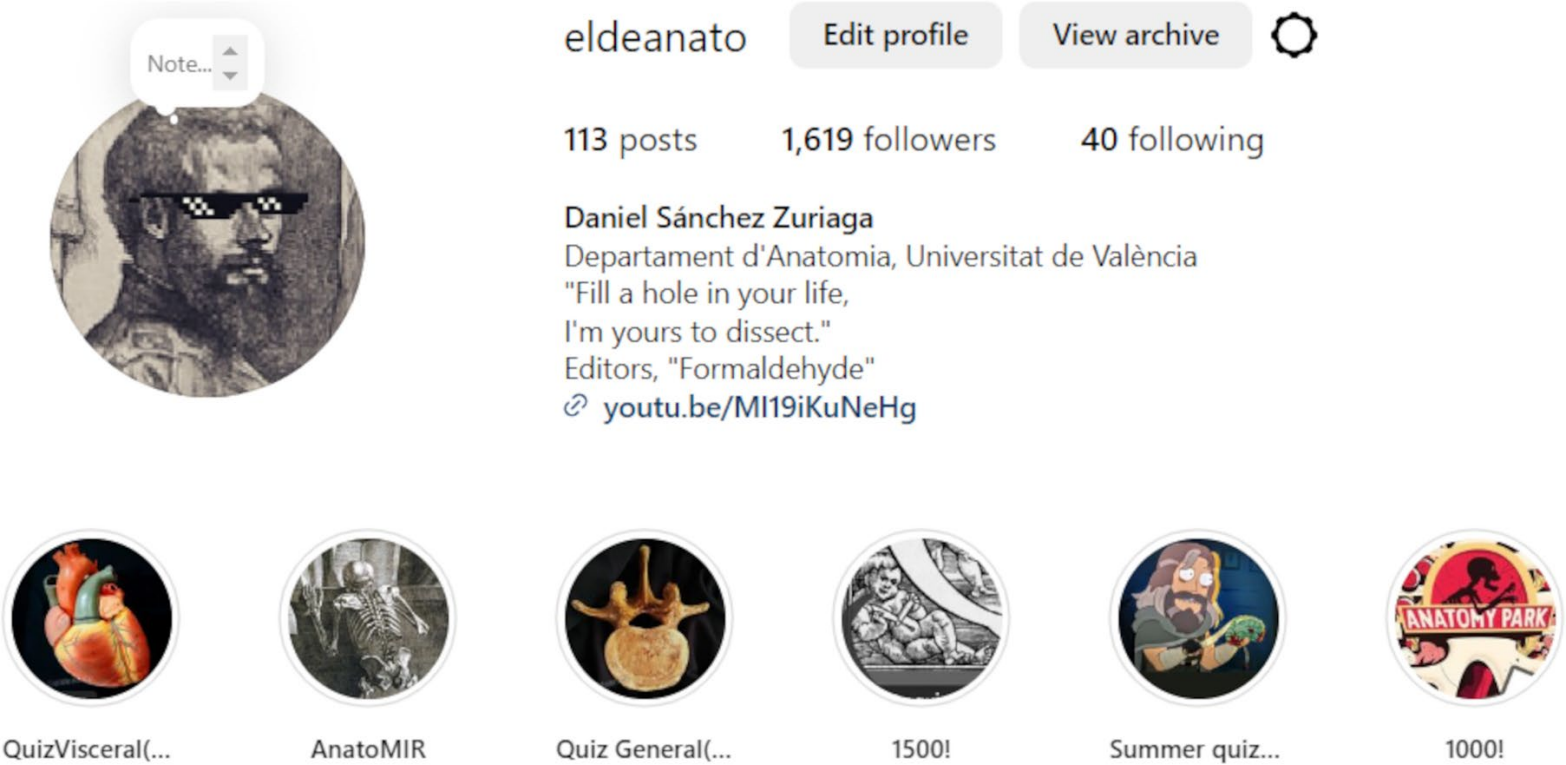
Instagram profile @eldeanato, with number of followers, number of posts and highlighted stories as of June 1, 2024

**Fig. 3:**
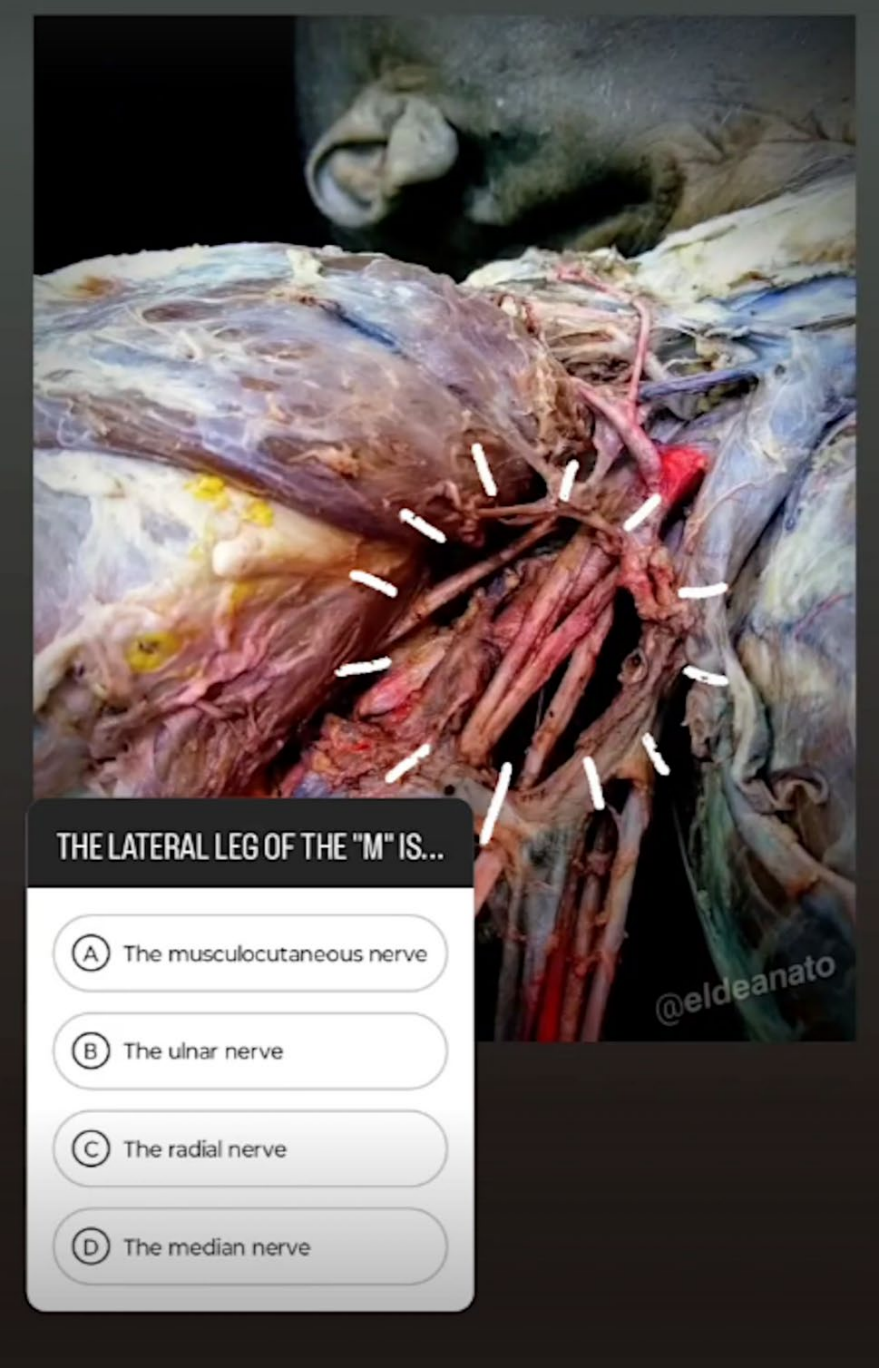
Instagram profile @eldeanato. Example of one of the questionnaires, with one of the newly created dissection images

Interactive resources were developed as an Instagram profile, @eldeanato (Fig. 2). The profile was overseen by a teacher from the Department of Anatomy, with a link to his professional profile included in the profile header. Instagram posts were based on anatomical illustrations and new photographs of dissections, with a description of the images and allusive quotes from literary works, songs or movies, in all the official teaching languages of the institution. Image captions included an announcement about the creation of a questionnaire during the following days, encouraging viewers to study the course contents related to the image. Questionnaires and images were uploaded to the profile coinciding with the cadaveric dissection practical sessions dedicated to the regions shown in those same images, so that students could interact with it in parallel to the upper limb anatomy classes. Quizzes were carried out weekly and were made using the questionnaire sticker of Instagram stories (Fig. 3).

Although the profile was kept public, the teacher managing it especially publicized the creation of the account among the 83 students of the group he was teaching that year. These 83 students were again explicitly told that their grades would not be influenced whatsoever by their amount of engagement with the profile. Nevertheless, given that, by its very nature, the interactive intervention required greater involvement, more time dedication and closer interaction with the students than the unidirectional intervention, and that its content was less in-depth and of a more playful nature, with a less obvious relationship with the content to be assessed in the exam, it was considered more appropriate in this case that the responsible professor would be the one in charge of teaching anatomy to the group.

#### Photographic Materials

All photographic materials used on these innovation projects were taken and processed by the teaching team responsible for the anatomy courses, from the cadaveric dissections used for the prosection practical classes. To maintain anonymity, precautions were taken in the publication of all photographic images to prevent the identification of the donors. Facial features, tattoos, and any other identifying characteristics were concealed. The cadaveric material came from donors who during their lifetime had signed a consent form for the donation of their bodies for educational or research purposes to the Department of Anatomy and Human Embryology of the University of Valencia. The donation process adhered to the recommendations of the Spanish Anatomical Society [22].

#### Satisfaction Questionnaires

The level of satisfaction of the students with both interventions, unidirectional and interactive, was subjectively assessed by means of an online survey, created using Google Forms. Both surveys were made up of four items, which measured the students’ use of the tool, the appeal of the intervention and the students’ perception of knowledge acquisition and motivating effects (Table 1). Both satisfaction surveys were very similar. Each was specifically given to the students in the group corresponding to each of the two interventions, immediately after the final exam of the Musculoskeletal Anatomy course, before publishing their grades so that they would not bias the students’ perception of knowledge gain. In this way, the students could globally assess whether they had gained knowledge with the intervention, once they had studied the complete syllabus for the exam, with all the tools at their disposal. Both questionnaires had four items, answered yes/no or scored using a five-point Likert scale (1, not at all; 2, slightly; 3, moderately; 4, very; 5, extremely).

**Table 1:** Satisfaction surveys

1) Use of the intervention	Have you entered the @eldeanato Instagram profile? / Have you entered the www.uv.es/sanzuda website?
2) Appeal of the intervention	Did you like @eldeanato Instagram profile? Did you like the www.uv.es/sanzuda website? Do you think it would be a good idea to implement this kind of intervention in other courses?
3) Perception of knowledge acquisition	How much would you say this activity has improved your knowledge of the subject?
4) Motivating effects	How much would you say this intervention has stimulated you to better prepare for your anatomy classes (Instagram profile)/the final exam (online lessons)?

### Web Analytics Tools

#### Google Analytics

The level of engagement of students with the unidirectional material was assessed using Google Analytics, a widely used and freely available web analytics service provided by Google. Google Analytics offers aggregated information of web usage data, such as the geographical origin of the website users, the duration of their session, the time spent by the user on each page, pages accessed per session or the bounce rate (the percentage of users who visit only the homepage without further exploring the website contents). Google Analytics has been previously used in a few recent studies to measure the effectiveness of educational innovation interventions in health sciences [23, 24], although none of them was related to anatomy courses.

In order to implement Google Analytics, its Java Script tracking code was added to every page of the website generated by eXeLearning. This code loads specific tracking files from the Google web servers which send web usage data to the Google Analytics user account (Fig. 1).

#### Instagram Engagement Metrics

There are several methods available to measure engagement on Instagram. Conventional approaches involve metrics such as the number of followers, the number of “likes” per post or the number of views of a story. However, these measures have limitations and may not always correlate with meaningful user engagement. For instance, a follower may not revisit the profile after following it or quickly scroll through stories without thoroughly reading them.

In our study, a more reliable method was used to measure engagement: the percentage of answers to the questionnaires in the stories. Calculating the number of questionnaire answers as a percentage of the total story views indicates users who not only viewed the story but also took the time to understand the question and provide an answer. In this regard, the percentage of answers to the questionnaires offers similar insights, but in the opposite direction, to the bounce rate provided by Google Analytics.

In order to make the two interventions, the Instagram profile and the online lessons, more comparable, both were limited to the same block of lessons, the ones about upper limb anatomy. To this end, although the Instagram profile remained active after the upper limb anatomy classes had finished, the percentages of answers to the Instagram questionnaires were calculated only on the quizzes related to this block of lessons. These questionnaires were published during the same weeks in which the students attended the theoretical and practical classes about upper limb anatomy.

Approval for this study was obtained from the university Consulting Committee on Teaching Innovation Proposals, with approval number/ID UV-SFPIE_PID-2078036.

## Results

### Web Analytics

As of the time of the final exam, the online unidirectional lessons about upper limb anatomy had 102 individual users. Users spent a relatively short time interacting with the material, with an average session duration of 2 min and 54 s. The bounce rate was high, at 38.6%.

The upper limb anatomy questionnaires in the @eldeanato profile received 654 ± 20 visits (mean ± standard deviation), with 274 ± 42 answers, resulting in an active engagement rate of 42%. This is to say, more than 40% of the people who viewed the stories answered the questionnaires. To date, the @eldeanato profile has more than 1600 followers with students from different countries, courses and biomedical areas and an average participation degree of approximately 30% in the story questionnaires.

### Satisfaction Surveys

Forty-one students answered the Google Forms satisfaction surveys, out of 83 enrolled to the Instagram intervention group, and 29 students out of 76 enrolled to the unidirectional website group. The results of both surveys were similar:

1. Use of the intervention: 100% of respondents in each group had entered the Instagram profile or the unidirectional website.
2. Appeal of the intervention: 97.6% of respondents in the Instagram profile survey, and 89.7% in the unidirectional website survey, rated this item with a 4–5 score in the Likert scale.
3. Perception of knowledge acquisition: 90.2% of respondents in the Instagram profile survey, and 93.1% in the unidirectional website survey, rated this item with a 4–5 score in the Likert scale.
4. Motivating effects: 92.7% of respondents in the Instagram profile survey, and 89.7% in the unidirectional website survey, rated this item with a 4–5 score in the Likert scale.

## Discussion

The present study attempted to evaluate the success of two types of innovative teaching interventions, an interactive Instagram profile and a unidirectional website. Results were different depending on the assessment tool used. According to satisfaction surveys, students were highly satisfied with both interventions. However, the results of web analytics measurements showed differences between both.

The interaction percentages observed in the Instagram story questionnaires were substantial, with 42% of viewers meaningfully interacting with the questionnaires. This degree of engagement is significant, as a 5% engagement level is considered high on Instagram [25]. It is true that the Instagram profile was being promoted by an active teacher of the group, but it is important to note that the active teacher promoted the content only to the 83 students in his group, and yet, the Instagram quizzes on upper limb anatomy were visited by an average of 654 viewers. These figures show that the engagement rate of the Instagram profile was very high, regardless of the effect of the teacher’s recommendations.

Using likes, shares, comments and followers as the sole measure of the success of Instagram content is problematic, as these engagement metrics are subjective [26] and come from Instagram posts, instead of stories. Instagram stories, although ephemeral in nature, tend to generate a much greater level of engagement on a daily basis [27]. We believe the participation rate in the Instagram stories questionnaires could be a reliable indicator of engagement, especially for educational interactive contents.

In contrast, students’ engagement with unidirectional materials was substantially lower, despite coming from a reliable source, the teaching staff from the Department of Anatomy in the medical school where the target group studied. The website did not spread much beyond the initially contacted 76 students, with a total of 102 users. It is important to consider that if the same person accessed the material from two different IP addresses (for example, their computer and their mobile phone), they were counted as two distinct individual users by the Google Analytics tracking tools. Almost every user was located in the same country and province as the University of Valencia. The interaction time was minimal, with an average session duration of less than 3 min, which does not even allow for a superficial review of the material. The bounce rate, almost 40%, shows how a significant portion of users only accessed the website’s homepage, which lacked content and served merely as an index for the rest of the pages.

Anatomy students face a content overloading issue, with an overabundance of information sources [28]. Several studies have observed that information overload is an actual problem for undergraduate anatomy students [18, 29]. It is possible that our website, no matter how reliable its contents may have been, was just one more among the many diverse sources of anatomical information that students use (class notes, material uploaded to virtual classrooms, textbooks, other web pages etc.) and did little beyond contributing to student’s information overload. This would explain the low rate of student engagement with this type of intervention.

However, satisfaction scores were high for both interventions. Teaching innovation interventions are usually assessed by means of satisfaction surveys, but they have many limitations. For a sample to be representative, the survey’s response rate must be high [30], but most teaching innovation satisfaction surveys have difficulty achieving response rates higher than 50%, as was the case in the present study. Teaching innovation satisfaction surveys also show a bias in the attitudinal composition of samples obtained, given the systematic differences between survey responders and non-responders. This causes a response bias, where the most satisfied users are the ones most likely to respond to the survey [31]. And there is also the so-called “ingratiating response bias” [32], the reluctance to criticize caregivers, health professionals or teachers when responding to a satisfaction survey. These biases may significantly impact the results of teaching innovation satisfaction surveys, leading to an overestimation of the level of satisfaction in the students. This could explain the contradictory results we have found between satisfaction surveys and objective measures of web engagement in the case of the unidirectional intervention, whose satisfaction levels may have been overestimated. Satisfaction studies have many inherent limitations and should not be used uncritically to assess the results of teaching innovation projects. Although satisfaction surveys have a role to play, they should be used with caution, and preferably as part of an array of assessment tools, which should always include objective engagement measures.

One of the biggest concerns about social media in general and Instagram interventions in particular is the lack of veracity and reliability of the contents [33]. To prevent the spread of inappropriate information, content creation should be at least supervised by educators and teachers. In the area of anatomy, the greatest concern is the dissemination of cadaveric images, which must have an educational purpose, as agreed in the informed consent for body donation. Exposing cadaveric material images without proper care may cause a bad impression on the general public, damaging the reputation of the institution and discouraging body donation. Each anatomy-related social media profile should have in its description the contact information of the teachers responsible for the project and the educational centre to which they are affiliated, which should allow the public access to their procedures and forms for body donation [22]. All the materials generated in the two interventions analysed in the present study met these requirements.

In summary, interactive interventions based on Instagram questionnaires seem to generate a much higher degree of engagement in anatomy students than traditional unidirectional interventions based on online lessons. This suggests that Instagram could be a useful tool to connect with anatomy students. In addition, it is relatively easy to use, and once a sufficient number of images of anatomical structures have been collected, it does not require excessive time for the creation and maintenance of the profile. However, the creation of new passive online materials is time-consuming, since in addition to the images and their description, it requires the writing of developed and verified texts, which also have to be kept up to date. Given the low engagement generated by this type of content, our results suggest that in areas of knowledge such as human anatomy, with an overabundance of information sources available to students, it may not be worth the investment of time necessary to create and maintain this type of resources.

Our work focuses on student satisfaction and web analytics parameters and does not include an analysis of learning results. The aim of social media interventions is not necessarily the direct enhancement of student learning. Arnbjörnsson [34] observed that interventions on social media do not appear to improve students’ grades, raising questions about why students value social media interventions so highly despite the lack of significant improvement in their academic performance. Kaczmarczyk et al. [35] suggested that this may be due to changes on the attitude towards the course subjects, which appear as more accessible and enjoyable when introduced interactively through social media. Nevertheless, the analysis of the possible effects of Instagram-based resources in anatomy learning results merits future investigations.

## Conclusions

An interactive educational innovation intervention based on Instagram stories questionnaires achieved a high degree of engagement among human anatomy students. On the contrary, a unidirectional intervention based on the publication of theoretical contents through a website achieved a much lower degree of engagement. These results contrast with the high degree of satisfaction generated by both interventions, measured through satisfaction surveys. The results of this study call into question the exclusive use of satisfaction surveys to evaluate the success of educational innovation interventions and support the use of Instagram as a teaching innovation tool in anatomy.

## Data Availability

Most of the data used for this study are on the text, and datasheets are available from the corresponding author upon reasonable request.
